# Pathological regression of primary tumour and metastatic lymph nodes following chemotherapy in resectable OG cancer: pooled analysis of two trials

**DOI:** 10.1038/s41416-023-02217-x

**Published:** 2023-03-25

**Authors:** Avani Athauda, Matthew Nankivell, Rupert Langer, Susan Pritchard, Ruth E. Langley, Katharina von Loga, Naureen Starling, Ian Chau, David Cunningham, Heike I. Grabsch

**Affiliations:** 1 Department of Gastrointestinal Oncology and Lymphoma, The Royal Marsden NHS Foundation Trust, London, UK; 2 Medical Research Council Clinical Trials Unit, University College London, London, UK; 3 Klinisches Institut fur Pathologie und Molekularpathologie, Kepler Universitatsklinikum, Linz, Austria; 4 Department of Pathology, Wythenshawe Hospital, Manchester University NHS Foundation Trust, Manchester, UK; 5 Department of Pathology, GROW School for Oncology and Reproduction, Maastricht University Medical Center, Maastricht, The Netherlands; 6 Division of Pathology and Data Analytics, Leeds Institute of Medical Research at St James’s University, University of Leeds, Leeds, UK

**Keywords:** Gastric cancer, Tumour biomarkers, Oesophageal cancer

## Abstract

**Background:**

No definitive largescale data exist evaluating the role of pathologically defined regression changes within the primary tumour and lymph nodes (LN) of resected oesophagogastric (OG) adenocarcinoma following neoadjuvant chemotherapy and the impact on survival.

**Methods:**

Data and samples from two large prospective randomised trials (UK MRC OE05 and ST03) were pooled. Stained slides were available for central pathology review from 1619 patients. Mandard tumour regression grade (TRG) and regression of tumour within LNs (LNR: scored as present/absent) were assessed and correlated with overall survival (OS) using a Cox regression model. An exploratory analysis to define subgroups with distinct prognoses was conducted using a classification and regression tree (CART) analysis.

**Results:**

Neither trial demonstrated a relationship between TRG score and the presence or absence of LNR. In univariable analysis, lower TRG, lower ypN stage, lower ypT stage, presence of LNR, presence of well/moderate tumour differentiation, and absence of tumour at resection margin were all associated with better OS. However, the multivariable analysis demonstrated that only ypN, ypT, grade of differentiation and resection margin (R0) were independent indicators of prognosis. Exploratory CART analysis identified six subgroups with 3-year OS ranging from 83% to 22%; with ypN stage being the most important single prognostic variable.

**Conclusions:**

Pathological LN stage within the resection specimen was the single most important determiner of survival. Our results suggest that the assessment of regression changes within the primary tumour or LNs may not be necessary to define the prognosis further.

## Background

The management of patients with resectable oesophagogastric cancer (OGC) consists of a multimodality approach usually combining systemic chemotherapy and surgery, which improves survival compared to surgery alone [1]. Despite this approach, current 5-year overall survival is only 45% [2], highlighting the urgent need to improve current treatment regimens and patient selection.

The United Kingdom (UK) Medical Research Council (MRC) OE05 clinical trial [3] was an open-label, randomised phase III trial in which patients with resectable oesophageal or junctional adenocarcinoma were randomly allocated to receive either two cycles of standard chemotherapy (cisplatin and 5-fluorouracil (CF) as proven effective in the earlier OE02 trial [4]), or four cycles of ‘experimental’ chemotherapy with epirubicin, cisplatin and capecitabine (ECX) followed by surgical resection. There were no differences in overall survival (OS), progression-free survival (PFS) or response rate (radiological and pathological) between the two arms. However, the frequency of good pathological response in the primary tumour was higher in patients treated with ECX chemotherapy.

The UK MRC ST03 clinical trial [5] was the successor trial to the UK MRC Adjuvant Gastric Infusional Chemotherapy (MAGIC) trial [1] which established perioperative epirubicin, cisplatin and 5-fluorouracil (ECF) as the standard of care for patients with resectable gastric or junctional cancer over surgery alone. ST03 was an open-label, randomised phase II–III trial assessing the safety and efficacy of the addition of anti-vascular endothelial growth factor (VEGF) monoclonal antibody bevacizumab to perioperative ECX chemotherapy. There were no differences in overall survival (OS), progression-free survival (PFS) or response rate (radiological and pathological) observed between the two arms.

In both trials, over half of patients who underwent resection died within 3 years of surgery. Thus, there is an urgent need to identify robust prognostic markers beyond the currently used pathological TNM staging to select patients who may benefit from additional or alternative therapy in order to prevent relapse.

The Mandard tumour regression grading system [6] is widely used in the assessment of response to neoadjuvant chemotherapy in OGC and is based on the comparison of the extent of fibrosis, presumed to be related to chemotherapy, to the extent of residual viable tumour. Although originally devised to determine prognosis following neoadjuvant chemoradiation in oesophageal squamous cell carcinoma, the Mandard scoring system has demonstrated prognostic value in OGC patients treated with neoadjuvant chemotherapy in univariate analysis [7]. However, multivariate analysis in previous studies of the MAGIC and OE02 trials demonstrated that the presence of lymph node (LN) metastases in the resection specimen was the only independent predictor of poor survival following neoadjuvant chemotherapy and surgery [8, 9].

The role of pathologically identified tumour regression changes within the LNs (LN regression (LNR)) has been investigated in retrospective analyses of single-centre cohorts with relatively small numbers of included participants. Some Western studies suggest that the presence of LNR is associated with improved survival [10–15]; whereas the only study published in gastric adenocarcinoma from Asia did not find additional value in determining LN regression over pathological LN status alone [16]. Considerable variation exists between these studies in terms of histopathological subtype (OG adenocarcinoma as well as oesophageal squamous cell carcinoma) and type of neoadjuvant treatment given (chemotherapy alone or chemoradiation). The assessment of LNR within previous studies was based on pathological features of tumour regression including fibrosis, acellular mucin pools and presence of certain cell types such as macrophages. In the absence of an internationally agreed pathological LNR grading system, there was variation in how LNR was scored which could, at least in part, explain the inconsistency of results seen thus far.

At the current time, there is no definitive evidence on whether the assessment of LNR in resection specimens after neoadjuvant chemotherapy allows patient prognostication or risk stratification. There is some data to suggest that heterogeneity exists in pathological regression changes between the primary tumour and metastatic tumour within LNs [13, 17] but the relationship with survival remains to be clarified.

The aim of this study was to investigate the prognostic role of regression within the primary tumour and metastatic lymph nodes, alone or in combination with other clinicopathological variables, in OGC resection specimens following treatment with neoadjuvant chemotherapy in a combined analysis of 1619 patients recruited into the OE05 and ST03 randomised clinical trials.

## Methods

### Study participants

Between January 2005 and October 2011, 897 patients were recruited to the OE05 trial and 794 of these (89% of randomised patients) underwent surgical resection. Between October 2007 and March 2015, 1063 patients were recruited to the ST03 trial and 929 of these (87% of randomised patients) underwent surgical resection. Based on the lack of improved response or efficacy with bevacizumab, patients who received this drug were also included in this analysis. Individual patient data were available from both trials for baseline demographics, clinical tumour and lymph node stage, pathological tumour and lymph node status and survival outcomes. These two studies were chosen as they represent the largest randomised trials conducted in this patient population in the United Kingdom and the clinical data and Haematoxylin and eosin (H&E) stained slides were readily available to our group. This study was approved by the UK national ethical approval system prior to study commencement (IRAS ID 257378).

### Assessment of tumour regression in the primary tumour and tumour within lymph nodes

All slides and/or blocks from ST03 and OE05 resection specimens were collected centrally. H&E-stained slides were scanned and reviewed by at least two independent senior pathologists with a special interest in gastrointestinal pathology who were blinded to the treatment arm. In cases of disagreement, a third senior pathologist reviewed the slides to establish the final score. Pathological tumour response in the primary tumour was determined according to Mandard [6]: TRG 1 (complete regression/fibrosis with no evidence of tumour cells), TRG 2 (fibrosis with scattered tumour cells), TRG 3 (fibrosis and tumour cells with a dominance of fibrosis), TRG 4 (fibrosis and tumour cells with a dominance of tumour cells), and TRG 5 (tumour without evidence of regression).

In the absence of an established scoring system for LNR, this was scored as either ‘present’ or ‘absent’ based on the presence of fibrosis, mucin lakes without viable tumour cells, large regions with sheets of foamy macrophages and the presence of relatively large areas of necrosis with or without tumour cells. The number of lymph nodes with or without regression was not taken into account for this assessment.

The clinical stage at diagnosis and pathological stage at resection were retrieved from databases held by the MRC Clinical Trials Unit at University College London, UK.

### Statistical analysis

The primary outcome measure for this analysis was overall survival (OS). OS was defined as the time from surgery until death from any cause, with surviving patients censored at their date of last follow-up. Factors considered in univariable analyses were Mandard TRG (1 vs 2 vs 3 vs 4 vs 5), clinical lymph node status (N0 vs N1 + ), pathological lymph node status (ypN0 vs ypN1 vs ypN2 vs ypN3, assessed using TNM version 6.0), LNR (present vs absent), and the combination of ypN status (ypN0 vs ypN1 or higher) and LNR (present/absent).

The Kaplan–Meier method and log-rank test were used to graphically present survival data and to estimate median survival and 3-year survival rates. The effect of each factor on OS was assessed using a Cox model to obtain hazard ratios (HR), without adjustment for any other covariates. Data were analysed using a two-stage approach: first analysing data per trial and then combining the data of both trials using a fixed-effects meta-analysis. Survival analyses included all trial participants who underwent surgery and had slides available for review. No imputation of missing data was performed. As these were hypothesis-generating analyses, no adjustment was made to account for multiple testing, and a *P* value of <0.05 was considered statistically significant.

To assess whether the available data could be used to define subgroups of patients with distinct prognoses, an exploratory classification and regression tree (CART) analysis was conducted. Variables considered for inclusion were pathological T category, pathological N category, grade of differentiation, resection margin status (R0 vs R1), LNR, the total number of lymph nodes and Mandard TRG. A test set was defined by taking a random sample of 70% of the patients, with the remaining 30% acting as a validation set. An additional split in the tree was added if a factor was significant with a *P* value of <0.01.

## Results

### Demographics

**Fig. 1:**
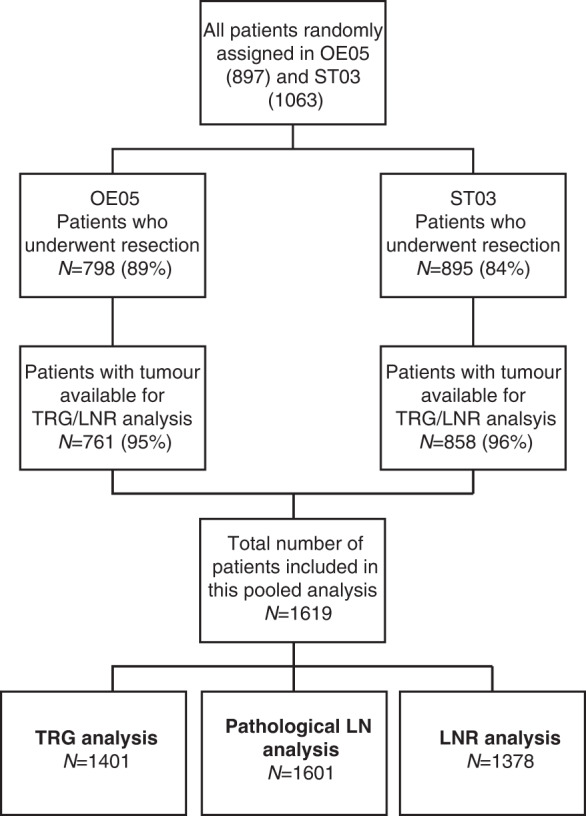
Consort diagram demonstrating the number of patients included in this analysis and sub-analyses. TRG tumour regression grade, LNR lymph node regression, LN lymph node.

From the OE05 trial, there were 19,489 H&E-stained slides available from 761 patients (96% of patients who underwent resection) for central review and assessment of TRG and LNR (390 (51%) from CF group and 371 (49%) from ECX group). From the ST03 trial, there were 20,277 H&E-stained slides available from 858 patients (92% of patients who underwent resection) for central review and assessment of TRG and LNR (440 (51%) from chemotherapy alone arm and 418 (49%) from chemotherapy plus bevacizumab arm). Once the two trials were combined, the total number of patients assessed within this analysis was 1619 (see Fig. 1), and their baseline characteristics are shown in Table 1. The median age of patients was 63 years (range 28–80 years) and 85% were male. Sixty-two percent of tumours were from the oesophagogastric junction (OGJ), 20% gastric and 15% lower oesophageal. The majority of tumours were clinically staged as T3 (84%) and node-positive (78%) at baseline. The patients included in this study were representative of the OE05 and ST03 study populations as a whole.

**Table 1:** Baseline patient characteristics of included patients from OE05 and ST03 trials.

	OE05 *N* = 761	ST03 *N* = 858	Total *N* = 1619
	No. (%)	No. (%)	No. (%)
Sex			
Male	685 (90)	693 (81)	1378 (85)
Female	76 (10)	165 (19)	241 (15)
Age			
Median (IQR)	62 (56–67)	63 (55–68)	63 (56–67)
Range	30–80	28–79	28–80
WHO performance status			
0	532 (70)	628 (73)	1160 (72)
1	229 (30)	230 (27)	459 (28)
Treatment arm			
CF	390 (51)	–	390 (24)
ECX	371 (49)	440 (51)	811 (50)
ECX + Bev	–	418 (49)	418 (26)
Clinical T-stage			
1	6 (1)	5 (1)	11 (1)
2	81 (11)	103 (13)	184 (12)
3	654 (86)	640 (81)	1,294 (84)
4	20 (3)	39 (5)	59 (4)
Clinical N-stage			
N0	164 (22)	180 (23)	344 (22)
N1+	592 (78)	604 (77)	1196 (78)
Tumour site			
Oesophageal	130 (16)	128 (14)	259 (15)
OGJ	610 (77)	459 (49)	1069 (62)
Gastric	–	342 (37)	342 (20)
Missing	53 (7)	–	53 (3)

### Primary tumour regression grade (TRG) and overall survival

**Fig. 2:**
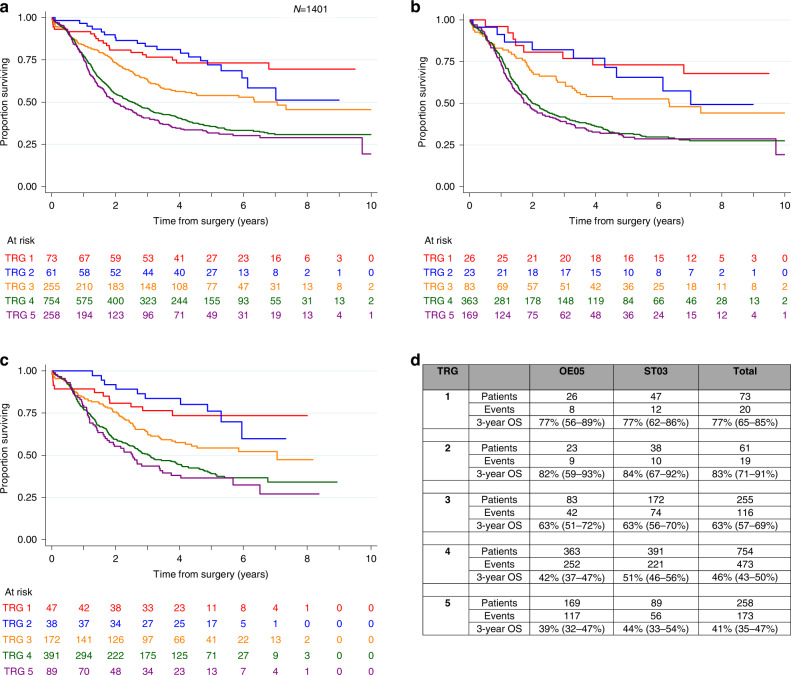
Overall survival by TRG grade. **a** All patients combined, **b** patients from the OE05 trial, **c** patients from the ST03 trial, **d** corresponding table with 3-year survival rates.

Of all tumours assessed centrally for TRG (*n* = 1401), 73 (5%) were classified as TRG 1 (complete pathological response), 61 (4%) TRG 2, 255 (18%) TRG 3, 754 (54%) TRG 4 and 258 (18%) TRG 5 (no evidence of regression). Patients with tumours with lower TRG scores had better survival than those with higher scores (HR 1.38 (1.28–1.50), *P* < 0.001; 3-year post-operative survival rates for TRG 1 77% (65–85%) vs TRG 5 41% (25–47%)). TRG scores of 1 and 2 were associated with the best survival, whilst TRG scores of 4 and 5 were associated with the worst survival, and TRG 3 tracked in-between, see Fig. 2.

### Lymph node status and overall survival

**Fig. 3:**
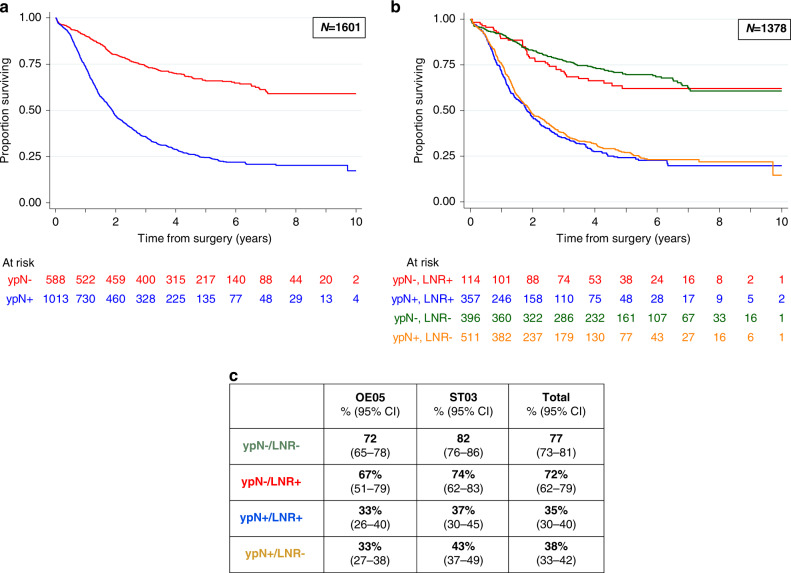
Kaplan-Meier survival curves. **a** Survival curves for pathological lymph node status (ypN-/ypN+). **b** Survival curves for pathological lymph node status with the presence or absence of lymph node regression (LNR+/LNR-). **c** Corresponding table to show 3-year survival rates for the four groups in graph **b**.

Within the OE05 trial, 188 patients (33%) of the 569 who were staged clinically as LN positive were staged LN negative (ypN0) at the time of resection. Of the 602 ST03 patients staged clinically as LN positive, 217 patients (36%) were staged as ypN0 in the resection specimen (see Supplementary Fig. 1 for more details). Patients with pathological LN negative disease at resection demonstrated the best survival, whereas those with LN positive disease at resection had the worst survival (3-year OS 74% (70–77%) vs 36% (33–39%), HR 3.4 (2.9–3.9), *P* = 0.0001, see Fig. 3a). All patients staged pathologically at resection as ypN0 had improved survival, regardless of whether they were staged clinically as LN positive or negative at baseline, compared to those with positive LNs (ypN1 + ) in the resection specimen (Supplementary Fig. 2).

### Tumour regression in lymph nodes (LNR) and overall survival

Three hundred and ninety-six (29%) of the 1378 specimens assessed for LNR were ‘true negative’ with no evidence of previous LN metastases. 3-year OS for these patients was 77% (73–81%). Of the remaining specimens, regressive changes in LNs were observed in a total of 471 (48%) cases (219 OE05 patients (46%) and 252 ST03 patients (50%)). A total of 22 patients (19%) staged clinically as LN negative at baseline had evidence of LNR in the resection specimen. 3-year OS for LNR present was 44% (40–49%) versus 38% (33–42%) for LNR absent (HR 0.85 (0.73–0.99), *P* = 0.0320.

LNR status was combined with pathological LN status (ypN) into four groups: those with negative LNs without regression (ypN-/LNR-), negative LNs with evidence of regression (ypN-/LNR+), positive LNs with evidence of regression (ypN+/LNR+) and positive LNs without evidence of regression (ypN + /LNR−) (see Supplementary Fig. 3). Figure 3b demonstrates that patients with pathological LN-negative disease (ypN-) had significantly improved survival regardless of whether they demonstrated evidence of LNR (72% (62–79%) 3-year OS) or not (77% (73–81%) 3-year OS). Conversely, those patients with pathological LN positive disease (ypN+ ) had poor survival regardless of whether they had evidence of LNR (35% (30–40%) 3-year OS) or not (38% (33–42%) 3-year OS). This finding was seen consistently within both trials individually and when combined.

### LN status, TRG and LNR by chemotherapy arm

Within the ST03 trial, there was no difference between the treatment arms (ECX vs ECX + bevacizumab) in terms of TRG score or presence of LNR (Supplementary Table 1). Within the OE05 trial, ECX was associated with a lower TRG score (TRG 1–2 12% vs 3%; *P* < 0.001) and higher rates of presence of LNR (38% vs 29%; *P* = 0.021) than CF. Neither trial demonstrated a correlation between TRG score and the presence or absence of LNR. There was also no significant difference in pathological LN negativity between the treatment arms in either trial.

### Classification and regression tree (CART) analysis

**Fig. 4:**
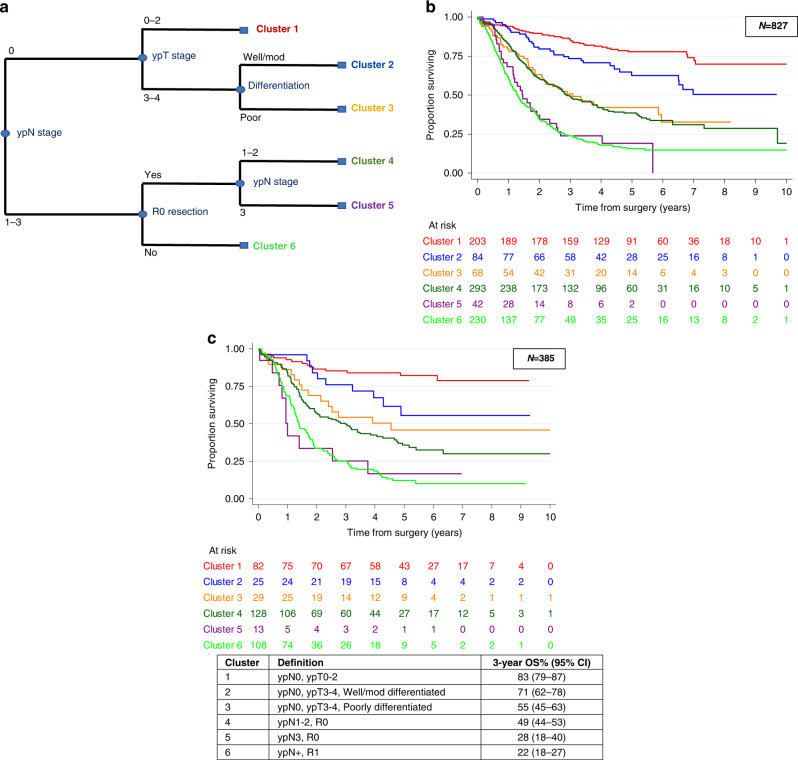
Exploratory CART analysis. **a** Split in CART analysis tree occurs if adjusted *P* < 0.01. **b** Kaplan–Meier survival curves for test set (70%). **c** Kaplan–Meier survival curves for validation set (30%) with the corresponding table of 3-year survival rates for each cluster.

In univariable analysis, lower TRG score, lower ypN stage, lower ypT stage, presence of LNR, well/moderate grade of differentiation after chemotherapy and absence of tumour at the resection margin (R0 resection) were all associated with significantly increased survival (see Supplementary Table 2). However, the multivariable analysis found that only ypN, ypT, grade of differentiation and R0 were independently related to prognosis. The exploratory CART analysis split the included variables into levels of prognostic importance and identified pathological N-stage (ypN0 vs ypN1–3) as the single most important variable for prognostication. Further variables considered prognostically important to cause a split in the tree were: pathological T stage, resection margin status and degree of differentiation. TRG score and LNR did not feature as prognostically important variables and hence did not cause a split in the tree (see Fig. 4a). Based on this exploratory CART analysis, six subgroups were defined with 3-year OS ranging from 83% (79–87%) for Group 1 to 22% (18–27%) for Group 6. The survival curves for these six subgroups within the test set are shown in Fig. 4b and the validation set in Fig. 4c.

## Discussion

In this study, we report the largest central pathology review and associations between LN metastases, TRG, LNR and survival in patients with resectable OGC treated with neoadjuvant chemotherapy and surgery within randomised clinical trials. We were able to assess pathological features in sufficiently large numbers of patients per tumour location (lower oesophageal, OGJ, gastric), which is important as current treatment strategies do not differentiate between tumour site.

The determination of TRG can inform clinicians about the chemotherapy sensitivity of the tumour, or at least subclones within the tumour, but is often prone to inter-observer variability. Our results for TRG support those seen in the central review of the OE02 trial [9] with TRG 1 and 2 demonstrating the best survival, TRG 4 and 5 the worst survival and TRG 3 in-between. However, although prognostic in univariable analysis, the relationship between TRG and OS is not independent of pathological LN status which was an independent predictor of survival following neoadjuvant chemotherapy and surgery in OGC. These results validate previous findings from studies in resectable OGC treated with neoadjuvant chemotherapy [8, 9] and highlight the importance of considering the optimal surgical procedure for individual patients to thoroughly resect LNs in order to accurately determine prognosis.

Our results also reinforce the current deficiencies in accurately determining LN staging at baseline as 22 (11%) of patients in our study were clinically staged as LN negative but demonstrated evidence of tumour regression within LNs in the resection specimen, indicating they were not truly negative to begin with. Staging assessment of LNs at baseline is a difficult task and currently relies on imaging and endoscopic ultrasound assessment, but the need to pursue a more accurate method may be less important, as we show that patients who were downstaged to ypN0 disease had similar survival to those with cN0 disease at the outset. Conversely, patients with residual tumour in LNs at resection had significantly worse survival regardless of their clinical LN status at baseline.

LNR in resection samples of oesophageal (both adenocarcinoma and SCC) and gastric cancers has been assessed. However, the neoadjuvant therapy within previous studies varied between chemoradiation and chemotherapy. This may influence results due to the effects of radiotherapy on the primary tumour bed which may enhance the locoregional response but, conversely, may undertreat micrometastatic disease. Two studies in oesophageal cancers (both adenocarcinoma and SCC) treated with chemoradiation prior to surgery proposed a LNR grading system based on histomorphological features in LNs (namely the degree of central fibrosis) and found the best prognosis in ypN0 groups with evidence of LNR in <3 LNs and worst prognosis in patients with residual metastases in >5% of resected LNs [10, 11].

Three studies of OG adenocarcinoma treated with neoadjuvant chemotherapy in Europe and Brazil [13–15] have demonstrated improved survival in patients with evidence of LNR who were downstaged to ypN0. However, the magnitude of survival benefit was akin to those patients who were deemed ‘true’ N0 i.e., negative LNs with no evidence of regression. Effectively, these findings are in keeping with the results of this present study. However, the presence of LNR was determined to be prognostic in these studies as it influenced survival in the node-positive groups, a finding which was not reproduced in our current study. A more recent single-centre UK study of 183 patients with gastric or OGJ type I and II adenocarcinomas who underwent neoadjuvant chemotherapy prior to surgery found that the survival of those with LNR was better than those with no evidence of LNR however not as good as those with true-negative LNs [18]. Conversely, a small study of 90 adenocarcinomas treated with chemotherapy +/− radiotherapy found that regression changes in LNs negatively impacted on survival and suggested that negative LNs with evidence of prior cancer should possibly count as positive pathological LNs in staging criteria [19]. The only study in Asia of 192 gastric cancers treated with neoadjuvant chemotherapy demonstrated that postsurgical T stage, R0 resection and ypN stage were independent predictors of survival [16]. Regardless of whether there were regression changes in the LNs or not, patients with residual tumours in LNs (ypN+) had significantly impaired survival compared to those with no residual tumour in LNs (ypN-). LNR was not an independent predictor of survival in multivariable analysis. These results are consistent with our findings in this UK population.

It is clear that considerable heterogeneity exists between these relatively small studies so no clear consensus on the assessment of LNR and its potential impact on prognosis has been possible thus far. Our results are therefore robust and important due to the number of included participants and the uniformity of their treatment (neoadjuvant chemotherapy alone) and histopathological diagnosis (all adenocarcinoma). The fact that the assessment of TRG and LNR was subject to central review by expert pathologists also guaranteed consistent classification of regression changes.

Previous studies which identified that response to chemotherapy is not homogenous between the primary tumour and LNs is in keeping with the results of this study as we found that there was no correlation between good regression in the primary tumour (TRG 1 and 2) and within the LN (LNR present). Given that evidence currently suggests there is more merit in clearing metastases within LNs in resectable OG adenocarcinoma, further work should focus on the optimal ways for neoadjuvant chemotherapy to target the LNs as efficiently as possible. The finding that the addition of epirubicin within ECX potentially results in better regression within the tumour as well as LNs is interesting and could warrant further investigation into the mechanistic action of this drug in OG cancer.

Early engagement of the lymphactic system by solid tumours is a clinical hallmark of cancer. Whereas the initial view was of a passive, linear relationship between LN spread and metastatic progression [20], it is now hypothesised that a highly complex series of cellular, molecular and structural changes occur within tumour-draining LNs (TDLN) to enable a permissive environment for metastasis via suppression of host antitumour immunity [21]. It is thought that lymph-borne factors which travel from the tumour to the TDLNs can help establish this tumour-supportive microenvironment. Furthermore, it has been suggested that increased immune activation due to tumour-related antigens can lead to changes in LN size due to follicular hyperplasia and the proliferation of lymphocytes. A study in resected oesophageal adenocarcinoma has indeed demonstrated that the presence of large regional negative LNs in resection specimens is associated with improved survival [22].

Given the importance of achieving LN-negative disease in resectable OG cancer, novel and rational therapies which can target the TDLNs should be sought. This is especially important since there is emerging evidence to suggest that LN colonisation may play a critical role in the development of distant metastases by inducing tumour-specific immune tolerance [23]. With a better mechanistic understanding, the TDLN is now emerging as a possible key player in response to immune checkpoint blockade and a potential direct target. It has been shown that higher expression of haematopoietic PD-L1 exists in the TDLN compared to non-draining LNs thereby exposing itself as a critical site of antigen presentation and T-cell priming [24]. Pre-clinical models have suggested that administering anti-PD-1/PD-L1 antibodies via routes that enrich delivery to the unique microenvironment of the TDLN may have the potential to enhance responses to checkpoint inhibitor therapy [25, 26]. This is particularly important for patients with resectable disease who are treated neoadjuvantly whilst the TDLN is intact. Further understanding of the distinct mechanisms by which LNs enable metastatic tumour cell survival is clearly required, and this knowledge is key to therapeutic targeting in patients with resectable OGC. Importantly, the possibility of targeting the antigen-rich but immune-suppressed LNs appears to be an exciting strategy for immunotherapy moving forward.

The current AJCC 8th edition staging system for OGC [27] has introduced a ypTNM group for patients who have undergone neoadjuvant therapy, cementing the fact that a staging system should accurately represent the population of patients to whom it is being applied. However, LN negativity (ypN0) transgresses each staging group and the survival curves are less distinctive between groups, suggesting further refinement or a different prognostic tool, utilising the robust prognostic effect of pathological LN status, is necessary for resectable OGC. Our exploratory CART analysis was able to define six subgroups with differing 3-year OS based on the independent prognostic pathological variables included. The single most important variable was confirmed as pathological LN stage (ypN0 vs yPN1–3), followed by pathological T stage and resection margin status and then the degree of differentiation. The analysis suggests that the assessment of LNR and TRG is not necessary to define prognosis further. Prospective validation is required to determine whether this combination of routinely-defined pathological factors alone can define prognosis in resectable OG adenocarcinoma following neoadjuvant chemotherapy and surgery above the AJCC staging system alone. The question remains as to what to do with patients with poor prognostic features after resection. At the current time, there is no evidence to allow physicians to offer an alternative adjuvant treatment to these patients despite the high chance of significant morbidity for no survival benefit. The Checkmate 577 study recently demonstrated a disease-free survival benefit for patients who received adjuvant nivolumab following neoadjuvant chemoradiation and surgery in oesophageal and junctional cancer [28], but the role of immunotherapy in the perioperative setting remains unproven, especially in the context of substituting for adjuvant chemotherapy in patients with poor prognostic factors following resection. Prospective randomised trials currently recruiting (such as the EORTC VESTIGE trial; NCT03443856) will hopefully provide an answer as to whether these high-risk patients will benefit from an alternative therapy strategy after surgery.

There are some limitations of our study: the actual number of LNs with features of regression within each specimen was not quantified. This was due to practical issues as the types of samples sent centrally were not uniform in their completeness making it difficult to definitively know whether all LNs were received or not. In addition, we did not test other proposed LNR grading systems nor attempt to determine our own grading system for LNR. However, other studies [14] have also taken this approach to determine the presence or absence of regression, without further grading or categorisation, along with the presence or absence of LN metastases at resection, and this is in keeping with the recommendations of a proposed grading system for LN metastases in gastric cancer treated with neoadjuvant chemotherapy [29]. Given that our results have shown that the determination of LNR may not be necessary to define prognosis further than the assessment of pathological LN status at resection, the need for a more intensive grading system does not appear evident.

In conclusion, our study demonstrates that in patients with resectable OG adenocarcinoma treated with chemotherapy, including a fluoropyrimidine and platinum, prior to surgery, the lymph node status in the resection specimen is the single most important determiner of survival. The regression changes within the primary tumour or metastatic LNs do not appear to influence prognosis any further. It may be possible to use routinely-defined pathological features beyond TNM to determine robust prognostic groups in OGC treated with neoadjuvant chemotherapy and surgery. Further work to widen our understanding of the mechanisms by which tumour-draining LNs enable metastatic tumour cell survival appears to be key for therapeutic targeting in patients with resectable OG cancer to achieve long-term cure.

## Supplementary information

Supplemental figures and tables

## Data Availability

Data supporting the results reported in this manuscript can be obtained by contacting the MRC Clinical Trials Unit at University College London.
