# Supplementary material for: Pathological regression of primary tumour and metastatic lymph nodes following chemotherapy in resectable OG cancer: pooled analysis of two trials
Source: Br J Cancer. 2023 Mar 25;128(11):2036–43. doi: 10.1038/s41416-023-02217-x (PMC10206103; doi:10.1038/s41416-023-02217-x)
Supplement: Supplementary file 1 — Supplemental figures and tables [file 41416_2023_2217_MOESM1_ESM.docx]

**Supplemental figures and tables**

**Figure 1: Tree diagrams showing clinical and pathological LN status for patients in OE05 and ST03.**

**Figure 2: Kaplan-Meier overall survival curves for patients by clinical lymph node stage at diagnosis (N-/+) vs pathological lymph node stage at resection (ypN-/+).**


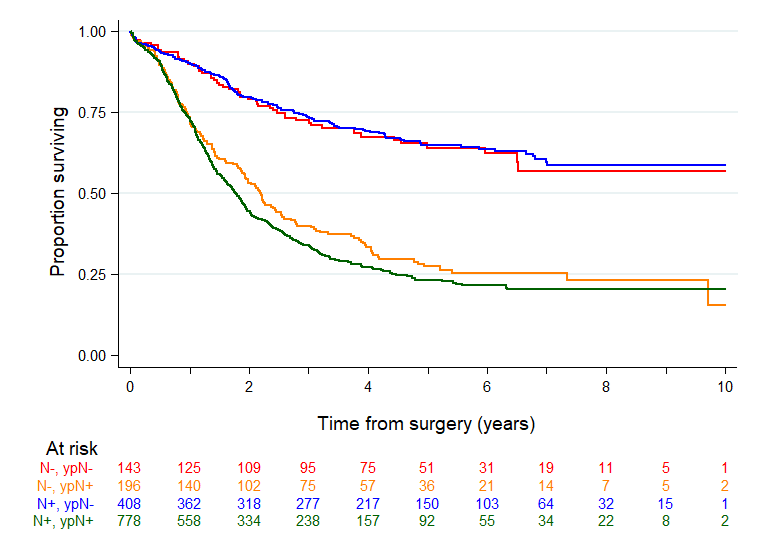


**Figure 3: H&E stained slides to show features of LNR and the corresponding ypN/LNR group each image belongs to.**

**B**


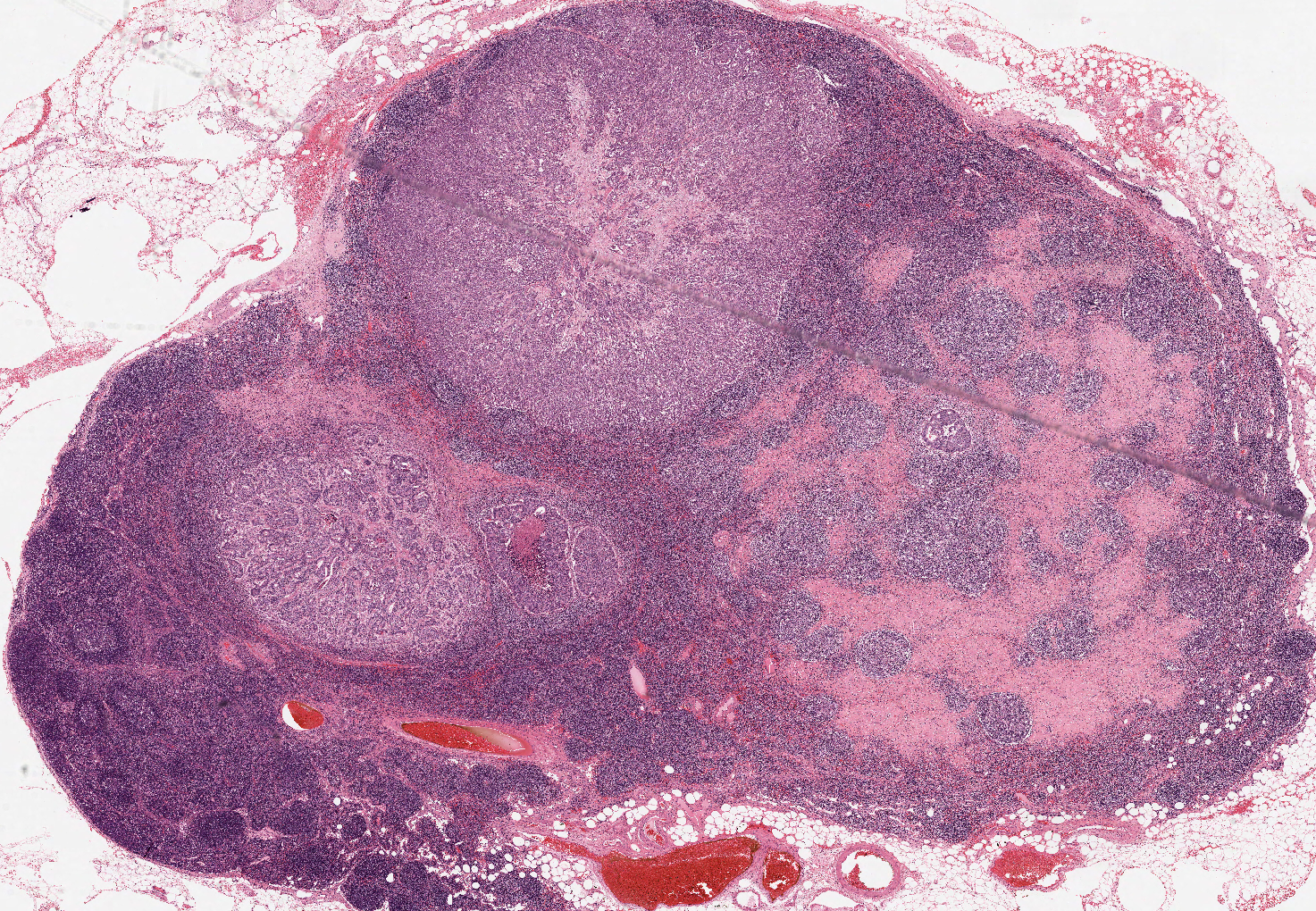


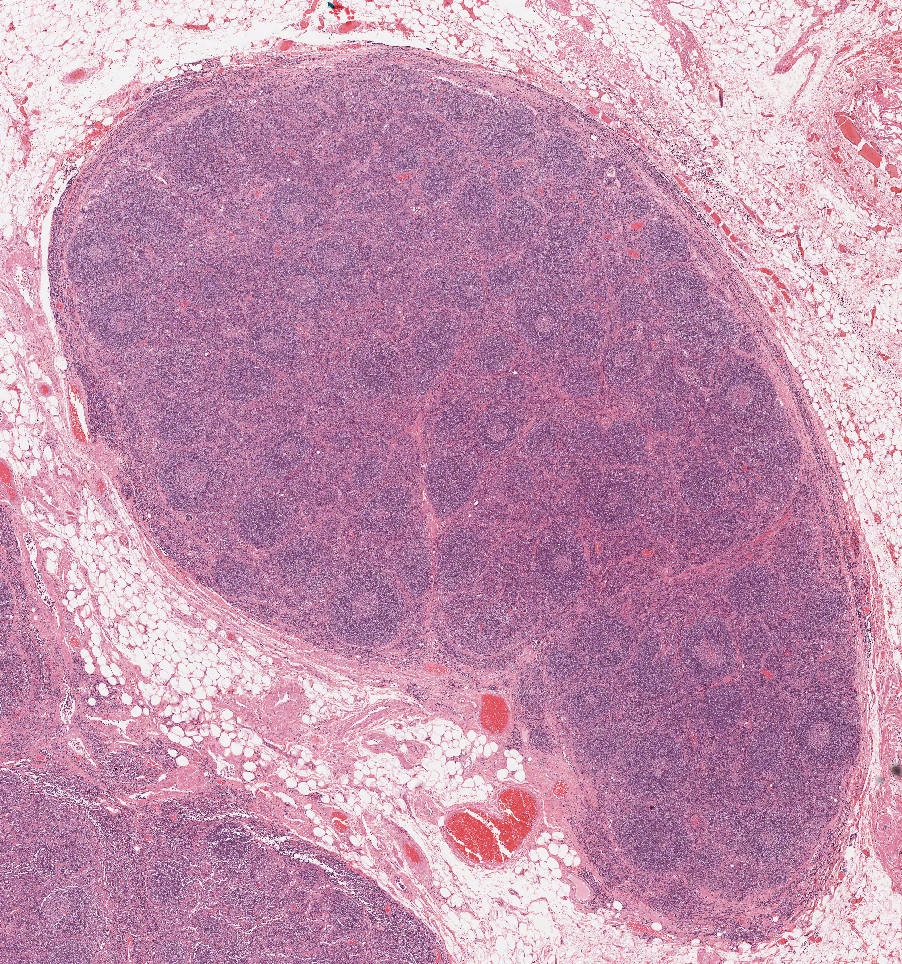


**C**

**A**


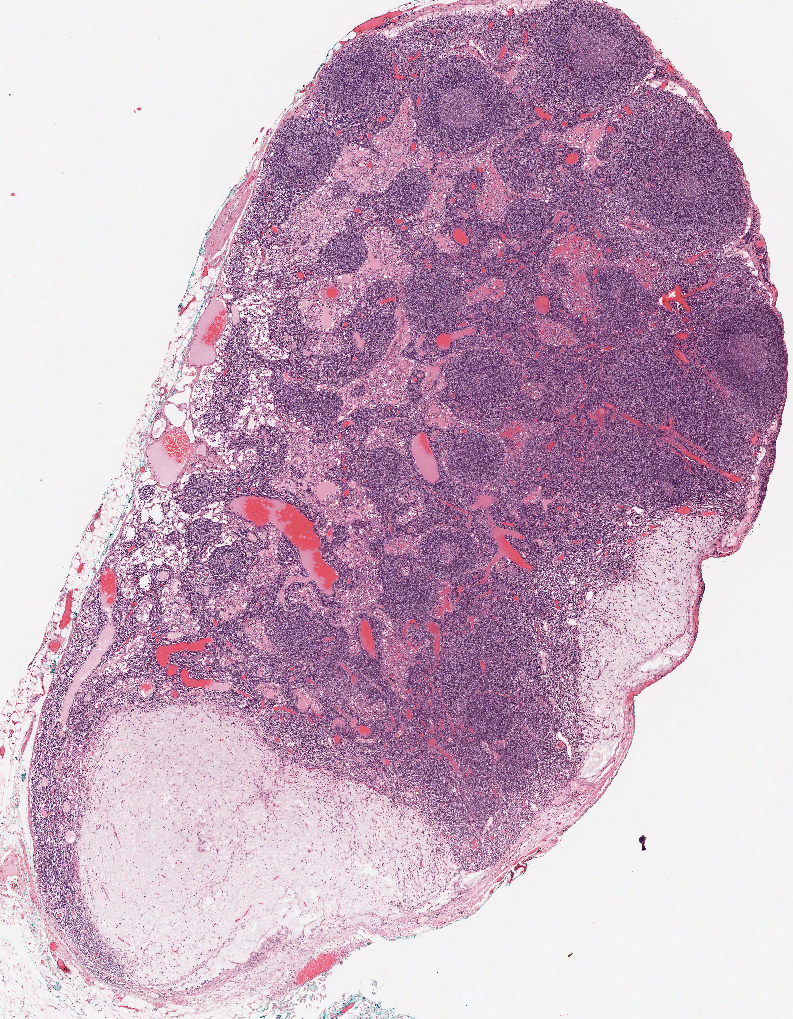


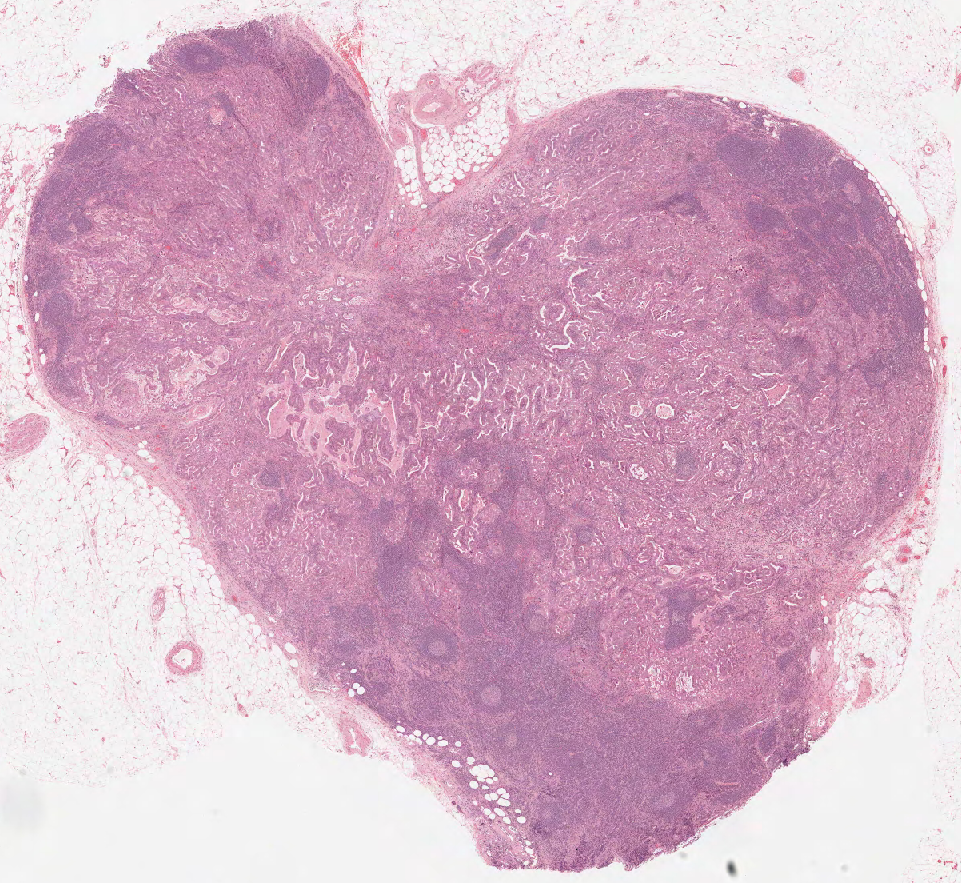


**E**

**D**


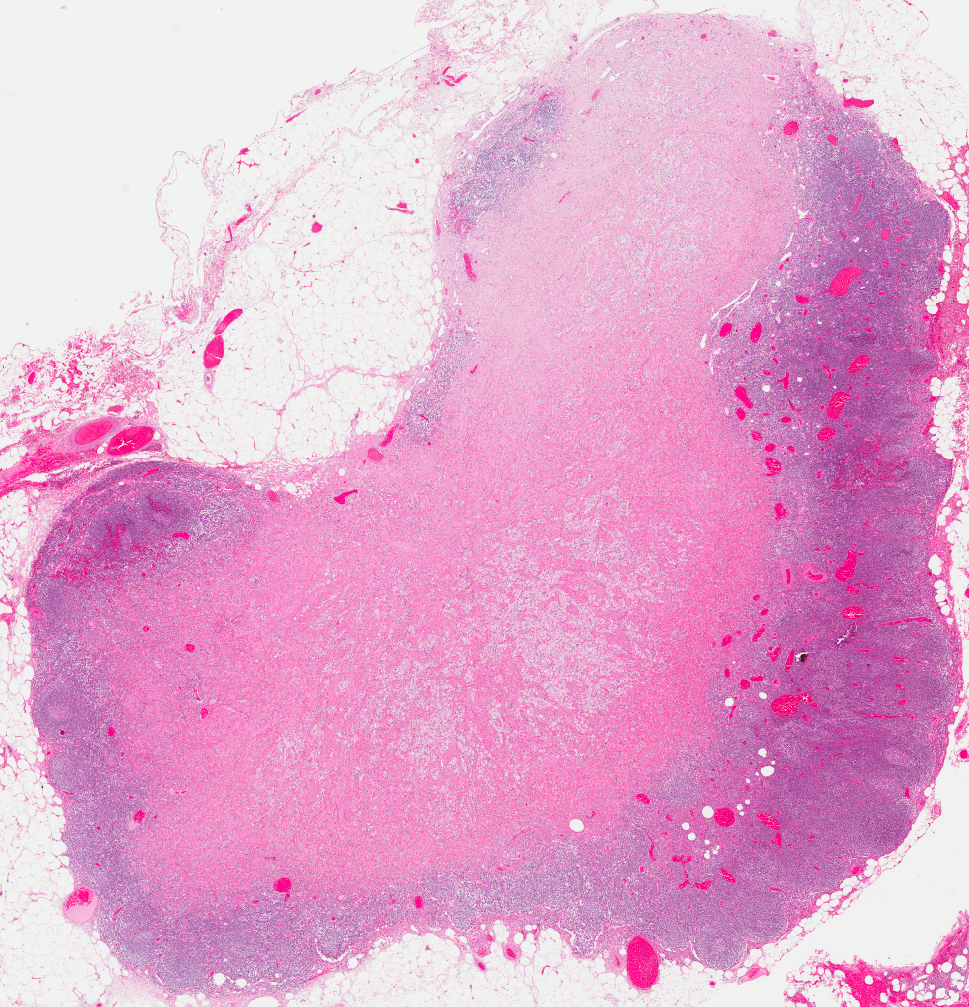


A

A . LN with no evidence of tumour and no regression (ypN-/LNR-); B. LN with tumour and signs of regression (pale pink areas) but with significant residual tumour (areas outlined in yellow) (ypN+/LNR+); C. LN with tumour and no evidence of regression (ypN+/LNR-); D. LN with signs of complete regression (acellular mucin pools outlined in green) , no residual tumour (ypN-/LNR+); E. LN with signs of complete regression (area of fibrosis outlined in blue, accumulation of foamy macrophages outlined in yellow), no residual tumour (ypN-/LNR+).

**Table 1. Pathological features by treatment arm**

|  |  | **OE05** | | **ST03** | |
| --- | --- | --- | --- | --- | --- |
|  |  | CF (%) | ECX (%) | ECX (%) | ECX + Bev (%) |
| **ypT stage** | 0 | 6 (2) | 19 (5) | 20 (5) | 24 (6) |
|  | 1 | 28 (7) | 50 (14) | 48 (11) | 47 (11) |
|  | 2 | 68 (18) | 61 (17) | 103 (24) | 85 (21) |
|  | 3 | 272 (70) | 225 (61) | 219 (51) | 215 (52) |
|  | 4 | 12 (3) | 11 (3) | 43 (10) | 41 (10) |
|  | | | | | |
| **ypN stage** | 0 | 116 (30) | 145 (39) | 176 (41) | 151 (37) |
|  | 1 | 234 (60) | 195 (53) | 123 (28) | 137 (33) |
|  | 2 | 28 (7) | 19 (5) | 72 (17) | 57 (14) |
|  | 3 | 10 (3) | 9 (2) | 61 (14) | 68 (16) |
|  | | | | | |
| **TRG** | 1 | 5 (1) | 21 (7) | 22 (6) | 25 (7) |
|  | 2 | 7 (2) | 16 (5) | 15 (4) | 23 (6) |
|  | 3 | 28 (8) | 55 (17) | 90 (24) | 82 (23) |
|  | 4 | 197 (57) | 166 (52) | 210 (55) | 181 (51) |
|  | 5 | 107 (31) | 62 (19) | 44 (12) | 45 (13) |
|  | | | | | |
| **Differentiation** | Well/moderate | 194 (51) | 189 (55) | 146 (36) | 153 (39) |
|  | poor | 188 (49) | 153 (45) | 260 (64) | 236 (61) |
|  | | | | | |
| **Resection margin** | R0 | 215 (60) | 227 (66) | 319 (74) | 306 (75) |
|  | R1 | 145 (40) | 115 (34) | 111 (26) | 103 (25) |
|  | | | | | |
| **LNR** | Present (+) | 100 (29) | 120 (38) | 130 (34) | 124 (35) |
|  | Absent (-) | 242 (71) | 198 (62) | 250 (66) | 232 (65) |
|  | | | | | |
| **ypN/LNR** | ypN-/LNR+ | 14 (4) | 29 (9) | 40 (11) | 31 (9) |
|  | ypN+/LNR+ | 85 (25) | 91 (29) | 88 (24) | 93 (26) |
|  | ypN-/LNR- | 86 (25) | 91 (29) | 118 (32) | 101 (29) |
|  | ypN+/LNR- | 155 (46) | 104 (33) | 126 (34) | 126 (36) |

**Table 2: Univariate analysis of impact of tumour factors on survival**

| **Factor** | **HR (95% CI)** | **p-value** |
| --- | --- | --- |
| Centrally reviewed Mandard TRG | 1.38 (1.28, 1.50) | <0.001 |
| Clinical N-stage | 1.13 (0.96, 1.32) | 0.147 |
| Pathological N-stage | 1.87 (1.74, 2.00) | <0.001 |
| Clinical T-stage | 1.06 (0.87, 1.29) | 0.591 |
| Pathological T-stage | 1.91 (1.74, 2.10) | <0.001 |
| LN regression | 1.36 (1.18, 1.57) | <0.001 |
| R0 resection | 2.52 (2.20, 2.88) | <0.001 |
| Differentiation | 1.64 (1.43, 1.81) | <0.001 |
| Total nodes resected | 1.00 (0.99, 1.00) | 0.555 |

All estimates obtained via meta-analysis of OE05 and ST03, through a Cox model with adjustment for age, sex, performance status and treatment arm. All significant factors were considered in CART analysis, plus sex and WHO PS. Total nodes resected was also included in CART in order to explore potential categorisations of the number of nodes.
